# Purinergic system in cancer stem cells

**DOI:** 10.1007/s11302-023-09976-5

**Published:** 2023-11-15

**Authors:** J. D. Nuñez-Rios, H. Ulrich, M. Díaz-Muñoz, C. Lameu, F. G. Vázquez-Cuevas

**Affiliations:** 1 Departamento de Neurobiología Celular y Molecular, Instituto de Neurobiología, Universidad Nacional Autónoma de México (UNAM), Boulevard Juriquilla #3001, Juriquilla Querétaro, Querétaro CP 76230 México; 2 Department of Biochemistry, Chemistry Institute, University of São Paulo (USP), São Paulo, Brazil

**Keywords:** Cancer stem cells, Purinergic receptors, Epithelial to mesenchymal transition, Ectonucleotidases, Purinergic signaling in cancer

## Abstract

Accumulating evidence supports the idea that cancer stem cells (CSCs) are those with the capacity to initiate tumors, generate phenotypical diversity, sustain growth, confer drug resistance, and orchestrate the spread of tumor cells. It is still controversial whether CSCs originate from normal stem cells residing in the tissue or cancer cells from the tumor bulk that have dedifferentiated to acquire stem-like characteristics. Although CSCs have been pointed out as key drivers in cancer, knowledge regarding their physiology is still blurry; thus, research focusing on CSCs is essential to designing novel and more effective therapeutics. The purinergic system has emerged as an important autocrine-paracrine messenger system with a prominent role at multiple levels of the tumor microenvironment, where it regulates cellular aspects of the tumors themselves and the stromal and immune systems. Recent findings have shown that purinergic signaling also participates in regulating the CSC phenotype. Here, we discuss updated information regarding CSCs in the purinergic system and present evidence supporting the idea that elements of the purinergic system expressed by this subpopulation of the tumor represent attractive pharmacological targets for proposing innovative anti-cancer therapies.

## The origin of cancer stem cells

Although cancer research has significantly progressed since the nineteenth century and several successful treatments have been developed since then, cancer is still a major cause of death worldwide. According to the World Health Organization, in 2020, cancer accounted for nearly 10 million deaths [1]. Currently, cancer is understood as a widely heterogeneous disease, and knowledge about intratumor complexity, which contributes to cancer progression and recurrence, therapy failure, and reduced overall survival, has been gained [2]. In 1855, Rudolf Virchow and Julius Cohnheim proposed that cancer results from the activation of dormant embryonic tissue remnants, giving rise to such a diverse population of cells. A modern interpretation of the observation made by those pathologists could be the cancer stem cell (CSC) model [3].

**Fig. 1:**
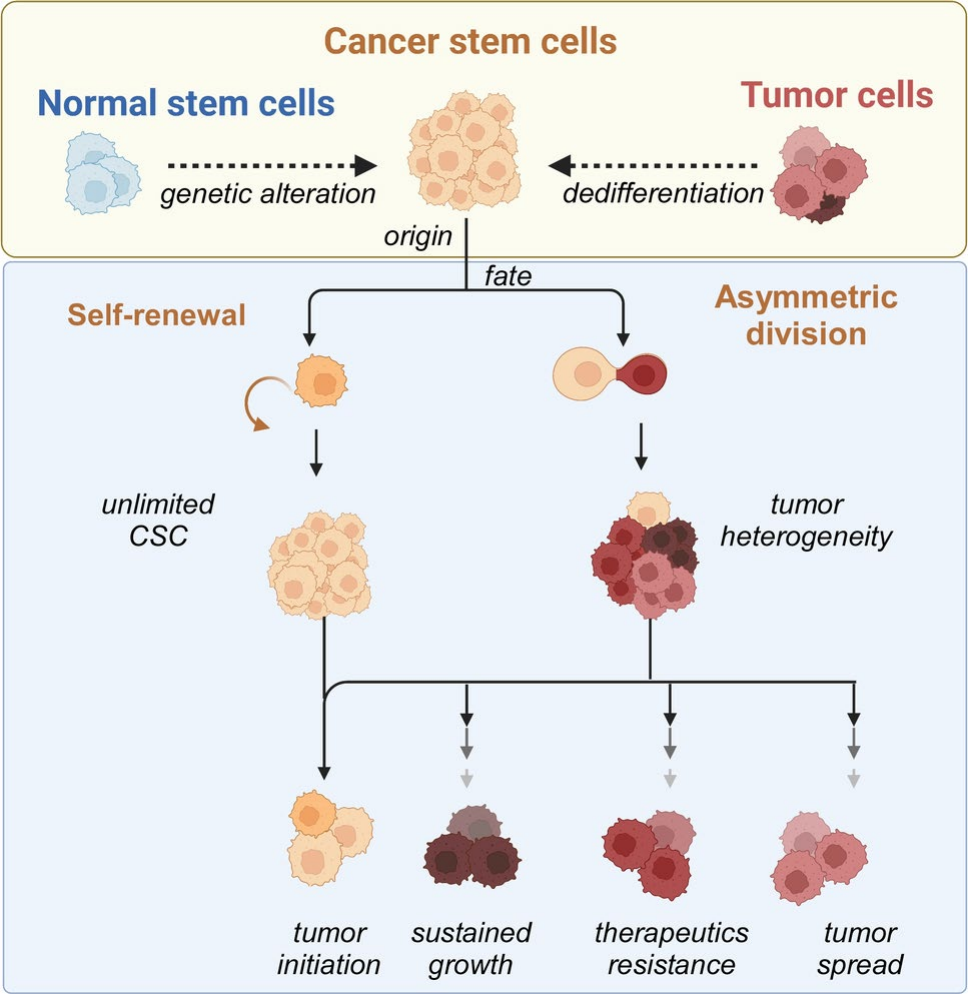
Origin and fate of cancer stem cells. Cancer stem cells are highly plastic cells in the tumor bulk. The hypothesis of their origin transits between two different proposals: that cancer stem cells originate from a genetic mutation of normal stem cells or that tumor cells are capable of dedifferentiating from a differentiated state to an undifferentiated state. Like normal stem cells, cancer stem cells can self-renew and divide asymmetrically, giving rise to an unlimited number of cancer stem cells or cells with compromised cell fates, respectively. Thus, cancer stem cells have the capacity to initiate a new tumor and generate phenotypically different tumor cells that sustain tumor growth, are resistant to current therapies, and have the motility to spread throughout the body and form secondary tumors. Created with BioRender.com

An important premise for the CSC model of cancer development is that the cells forming the tumor are phenotypically heterogeneous and hierarchically organized. This heterogeneity and hierarchy is supported by the differentiation grade of each cell with respect to an original ancestor [4]. In this hierarchy, notwithstanding the phenotypical diversity of the tumor tissue, only one type of cell has the competence to initiate cancer and is the source of each one of the phenotypes in a tumor; these are CSCs. CSCs display particular characteristics resembling normal stem cells (nSCs) (Fig. 1): (1) self-renewal potential to ensure the presence of tumor-initiating cells, a property that in nSCs is achieved by asymmetric cell division [5]; and (2) differentiation potential to give rise to the phenotypical diversity of a tumor [6]. In addition, the CSC subset of the tumor bulk also acquires resistance to conventional therapy, thus making them a possible cause of cancer recurrence [7].

CSCs were first identified in the experiments by Bonnet and Dick in 1997, where they proved that only a small subpopulation (0.1–1%) of acute myeloid leukemia (AML) could restore the disease after being transferred to immune-deficient mice, and subsequent propagation of the disease was also possible [8]. These cells (with the phenotype CD34^+^CD38^neg^) resembled those in the hematopoietic system. Similar observations were made for solid tumors in breast epithelial cancer. In a seminal work by Michael Clarke’s group, tumor cells were isolated according to their individual phenotypes and xenotransplanted into immunocompromised SCID mice. Clarke and colleagues found that only cells with a CD44^+^/CD24^low/−^ESA^+^ phenotype had the ability to induce a new tumor with full phenotypic diversity [9]; in turn, the tumor could be sequentially propagated. In parallel, human brain tumor-initiating cells were identified; in this case, it was demonstrated that only cells positive for the surface marker CD133 could originate cells with similar phenotypes to those observed in patients [10].

Thanks to these primordial studies, much effort has been focused on detecting CSCs in different tumor tissues, such as the ovary, prostate, lung, pancreas, neck and head, and colon [11–17], and many glycoproteins, including CD44, CD133, CD38, CD24, CD34, and CD73, have been linked to stem-like properties [18]. Different tumor markers have been identified depending on the type of cancer being studied. Hence, CSCs are not a single entity but rather a complex system with myriad phenotypes.

A question inherent to the CSC model is the origin of these tumor-initiating entities (Fig. 1). First, it was proposed that CSCs originate from nSCs through the accumulation of mutations in specific loci coding for tumor suppressors and/or oncogenes, resulting in malignant transformation [6, 19–21]. The CSC hypothesis has identified strategies for novel cancer cell treatment based on the stem cell theory, which involves a hierarchical organization from undifferentiated cells toward progenitors with restricted differentiation potential and finally terminal differentiation. Therefore, CSCs have greater oncological plasticity potential than more differentiated cancer cells. A comparison of CSCs with nSCs would provide common mechanisms and facilitate the treatment of recurrent tumor lapses, metastasis, and drug resistance. Nevertheless, untransformed nSCs and CSCs cannot be directly compared due to the complexity of their embryogenesis and stem cell development, as well as their epigenetic backgrounds. Furthermore, Teng and collaborators contest the CSC hypothesis and argue that tumor cells maximize their survival potential through a dedifferentiation process that occurs during microenvironmental stress (i.e., chemotherapy conditions) rather than through a hierarchical tumor development cascade [4]. In summary, interesting alternatives have been proposed, such as the acquisition of CSC properties by differentiated cells through different mechanisms, including gene transfer, environmental influence, and genomic instability [22, 23]. CSC phenotype acquisition is a fundamental question that may involve more than one mechanism and will therefore continue to generate controversy and require further investigation.

## The microenvironment for CSCs

Physiologically “normal” stem cells, here named nSCs, reside within specific conditions that maintain the stem-cell state; these include cell-to-cell and cell-to-extracellular matrix (ECM) interactions and signals that repress and activate diverse cell fate programs in order to preserve self-renewal and keep a dormant state. The “stem cell niche” was first proposed by Schofield in 1978 based on the observation that the spleen is unable to support the hematopoietic stem cell state in the same way that the bone marrow can, concluding that there are specific conditions (i.e., a microenvironment) that support the stemness and differentiation capability of stem cells [24]. Since then, several nSC niches have been identified in adult tissues such as the hematopoietic system, skin, intestine, brain, and muscle [25]. A niche is thus defined by its functionality rather than by its location, as nSC proximity to certain components of the microenvironment does not indicate that the stem state is supported.

As mentioned previously in this review, the tumor is a complex and heterogeneous entity containing not only cancerous cells but also resident stromal host cells, soluble factors, and the ECM, all of which favor tumor growth and progression [26]. Thus, it is not surprising to realize that there are niches within the tumor that support a stem-like phenotype. In a similar manner to nSCs, CSCs reside in niches within the tumor microenvironment (TME), preserving their phenotypic plasticity and conferring immune suppression and protection against chemotherapy [27]. Many cell types have been described to comprise the CSC niche, such as fibroblasts, endothelial cells, adipocytes, myeloid-derived suppressor cells (MDSCs), macrophages, and CD^4+^ T-cells, among others [28]. Altogether, these diverse cell types orchestrate a malignant phenotype, allowing chemotherapy and radiotherapy resistance, cancer initiation and recurrence, and metastatic capability. Cancer-associated fibroblasts (CAFs) have been linked to a tumor-supportive environment in different models. For example, in a head and neck cancer model, cancer cell-secreted transforming growth factor beta (TGF-β) augmented periostin, an ECM component, leading to proliferation, migration, and metastasis [29]. Similarly, in a breast cancer model, normal fibroblasts that were irradiated and injected into the mouse mammary fat pad overexpressed TGF-β and hepatocyte growth factor (HGF), thereby supporting tumor initiation [30].

Endothelial cells have also been associated with favoring a malignant phenotype in a prostate cancer model, where *human umbilical vein endothelial cells* (HUVEC) co-cultured with prostate cancer cell lines favored tumor initiation and metastasis, thus activating autophagy, which has been related to a stem-like phenotype [31]. In addition to these stromal cells, immune cells are recruited and re-educated in order to favor malignancy and stem-likeness. In an ovarian cancer model, CSCs isolated from the OVCAR-3 (a metastatic ovarian carcinoma cell line) interacted with macrophages polarized to an M2 phenotype, which diminished chemotherapeutic sensitivity and increased invasion in a transwell assay and tumor formation in a mouse xenotransplant model. These processes were thought to be ignited by WNT pathway overactivation [32].

The TME is not just a silent spectator but rather an active component within the tumor bulk. In addition to the previously mentioned cell types, the TME also contains non-cellular elements, such as the ECM, which in healthy conditions helps to maintain tissue structure and homeostasis but in cancer has been related to supporting malignancy and metastasis. The ECM can perpetuate a malignant stem-like phenotype, given that modifications in the ECM can alter fibroblasts, endothelial cells, and immune cell functions, which can further reinforce ECM modifications and prolong the malignancy [33]. Moreover, the ECM can provide an anchoring site for CSCs through their matrix-interacting receptors, including integrins, CD47, and CD44. The latter has been used as a CSC marker in several *in vitro* models and patient-derived samples. Its binding to hyaluronic acid (present within the ECM) has been proven to activate NANOG, thereby eliciting the expression of pluripotent stem cell regulators (e.g., *Rex1* and *Sox2*). CD44 has also been shown to interact with STAT-3, promoting multidrug-resistance transporter *MDR1* expression, which results in chemotherapy resistance [34].

Early in tumor development, there is reciprocal communication between the components of the TME and cancerous cells, promoting angiogenesis, nutrient supply, and waste removal. Many soluble factors constitute the TME, such as growth factors, hormones, and signaling molecules. TGF-β has been one of the most studied and well-characterized of these factors. TGF-β can have a dichotomous effect as it limits or preserves CSC populations. In gastric carcinoma, it was shown to decrease the side population by down-regulating ABCG2, an important chemotherapy efflux mediator [35]. On the other hand, several studies have proved that TFG-β facilitates the stem-like phenotype in solid tumors (e.g., breast cancer, glioblastoma, and leukemia) [36] by increasing CD133 and CD44 expression, thereby potentiating the stem-like populations. Other growth factors have been linked to promoting stemness in CSCs, such as epidermal growth factor (EGF) and fibroblast growth factor (FGF). In colon cancer, EGF proved to be essential for CSC selection and maintenance, and its inhibition promoted CSC apoptosis due to a decreased basal activation of Akt and ERK [37]. Conversely, FGF was demonstrated to induce a malignant phenotype in “healthy” induced pluripotent stem cells, where chronic exposure to this growth factor led to a CSC population that no longer required FGF in order to sustain its survival [38].

As previously stated, the TME includes soluble signaling molecules regulating the cancerous phenotype, and given that the purinergic system is ubiquitous and modulates many cell processes (e.g., proliferation, differentiation, and migration), ATP concentrations within the intra-tumor milieu can reach the millimolar range. In healthy tissues, they remain below the micromolar range, as Pellegatti and Di Virgilio elegantly demonstrated [39]. Extracellular ATP (eATP) is actively secreted into the extracellular space in a basal manner. Once there, ATP is metabolized to ADP, then AMP, and finally to adenosine (ADO) by the ectonucleotidases CD39 and CD73. Several stressors, such as hypoxic conditions (highly observed in necrotic tumors), acute and chronic inflammation (in the TME), and anti-cancer therapy-induced cell death, promote the accumulation of eATP and, thus, ADO. Within the TME, eATP and ADO seem to play contradictory roles since ATP is a “find me” signal, recruiting the immune system, and ADO is an immune suppressor [40]. Moreover, there is evidence suggesting that ATP can also induce immune suppressive regulatory T cell (Treg) expression through dendritic cell activation by chemotherapy-induced cell death [41]. Altogether, this information suggests that the primary function of the purinergic system, namely ATP and ADO, is purely related to the immune system; however, this could not be further from the truth, as we will argue in the next sections by explaining in detail the role of ATP in the TME.

## Nucleotide and nucleoside signaling

### Chemistry

Purines are an extended family of aromatic and heterocyclic molecules. 9H-purine, the simplest of these compounds, is formed by two rings: a pyrimidine fused with an imidazole. The alternating presence of single and double bonds among N and C atoms allows the formation of conjugated systems characterized by the coincidence of an overall low molecular energy and, at the same time, high molecular stability. The chemical underpinning of these properties is the existence of interatomic conjugated bonds connected by π-orbitals with delocalized electrons [42]. Different nucleobases, including purines, have been detected in carbonaceous meteorites [43]. In addition, it has been widely reported that adenine can be formed from simpler molecules, such as NH_4_^+^/NH_3_, CN^−^, CO, CH_4_, H_2_, and formamide, under abiotic primordial conditions [44]. These facts strongly suggest that purines, as well as many other “organic” molecules, were already present in early terrestrial times and were selected during the prebiotic evolution that occurred prior to the emergence of the first living entities on our planet [45].

The two principal purines with biological activities are adenine (6-amino-purine) and guanine (2-amino-6-hydroxypurine). In this review, we only address the importance and characteristics of adenine and its relationship with nucleoside and nucleotides in purinergic signaling through their actions on different membrane receptors. ADO contains a molecule of adenine attached to a ribofuranose moiety via a β-N (9)-glycosidic bond. ADO shows keto-enol tautomerism, being the keto form predominant at pH 7 [46]. In addition, the rotation of the glycosidic bond allows the presence of two ADO conformations: syn and anti. The *syn* conformation is favored in physiological conditions [47]. ADO is a polar molecule with a partition coefficient XlogP3 value of − 1.1, where negative coefficients are indicative of hydrophilic compounds. Its dipole moment has been calculated to be 3.93 Debye, showing an orientation toward the region between C6 and N3, and being located in the middle of the purine and sugar rings [48].

In contrast to ADO, ATP is a nucleotide with a negatively charged triphosphate moiety and a net electrical charge of − 4 at physiological pH. ATP and other nucleotide triphosphates form organic complexes with divalent cations, most commonly Mg^2+^. Mg^2+^ can coordinate with ATP^4−^ by either all three phosphate groups (C3 configuration) or by the terminal β and γ phosphate groups only (C2 configuration) [49]. Because of its ionic characteristics, the XlogP3 partition coefficient of ATP is clearly more negative (− 5.7) than the one calculated for ADO (− 1.1). Molecular dynamics simulation of ATP has suggested that the conformations of this nucleotide in water change drastically when ATP is bound to diverse proteins, especially in the C2 of the ribose ring, the adenine ring, as well as the torsion angles of the glycosyl bond and the bond between phosphate and ribose [50].

In addition to signaling through purine nucleotides and nucleosides, the UTP and UDP pyrimidines, pyrimidine dinucleotides (i.e., Ap4A), and sugar nucleotides (UDP-glucose and UDP-galactose) activate specific receptor subtypes (reviewed in [51]).

### Intracellular metabolism

ATP and ADO are part of a large set of interconnected purine intermediates that play diverse metabolic and signaling roles as extracellular and intracellular molecules by means of catabolic and anabolic transformations. These metabolic conversions are particularly relevant since only the liver and kidney are fully capable of synthesizing purines *de novo* from simpler units such as glycine, glutamine, aspartate, formate, and HCO_3_^−^ [52]. Hence, some tissues (e.g., brain and muscle) express the salvage pathway that allows the formation of purine nucleotides from metabolic intermediates like hypoxanthine. Therefore, purine transit is established between these tissues (receptors) and the liver (emitting source), with ADO and hypoxanthine in plasma and erythrocytes being the metabolic intermediates. This metabolic communication is subjected to circadian regulation [53].

ATP is a key factor in energy metabolism that controls intracellular electron fluxes from reducing nutrients to mitochondrial oxygen. In the sixties, Atkinson and Walton postulated that the proportion of adenine nucleotides, known as the adenylate energy charge ([ATP] + ½[ADP]/[ATP] + [ADP] + [AMP]), regulates the balance between anabolic and catabolic reactions [54]. This concept was further tested and ratified with the identification of a set of allosteric enzymes sensitive to the AMP/ATP ratio that modulates the biosynthetic and degradative metabolic pathways, as well as with the existence of energy sensors such as AMP-kinase [55]. ADO treatment is capable of increasing the hepatic energy charge *in vivo* by enhancing mitochondrial activity [56].

ADO plays various intracellular roles. ADO catabolic transformation results in uric acid in mammals without uricase, a free radical scavenger [57]. The redox enzyme that produces uric acid, xanthine dehydrogenase, can also act as an oxidase in Ca^2+^-promoted protein alteration. Xanthine oxidase has been proposed as a source of the free radical anion superoxide (·O_2_^−^) in pathological conditions such as inflammation and ischemia [58]. ADO can also combine with the sulfur-containing molecule homocysteine to form S-adenosyl-L-homocysteine (SAH). SAH is an inhibitor of methylation reactions that depend on S-adenosyl methionine (SAM). Therefore, ADO can indirectly modulate the formation of methylated intermediates and influence processes including neurotransmission, epigenetics, and membrane fluidity [59].

### Signal transduction

In addition to its well-known role in energy exchange reactions, ATP is an important signaling molecule with characterized release mechanisms and specific receptors. In 1929, researchers showed that adenine influences cardiac rhythm, thus indicating that ATP plays a role in extracellular signaling [60]. In 1972, Burnstock proposed ATP as a non-adrenergic and non-cholinergic neurotransmitter [61], giving rise to a new field of study: the purinergic system. At first, his proposals were not accepted by the scientific community but were gradually earning a place in the field thanks to the cloning of purinergic receptors that mediate the signal in response to extracellular purines. These receptors have been classified into two major types based on agonist selectivity: P1 adenosine receptors and P2 nucleotide receptors. The former are conformed by four receptors (A1, A2A, A2B, and A3), and the latter are divided into two main subtypes: P2X and P2Y receptors, which are ligand-gated ion channels and G-protein-coupled receptors, respectively [62].

To date, seven P2X subunits have been cloned in mammals (P2X1-P2X7), and these subunits can form either a homotrimer or heterotrimer that acts as a ligand-gated ion channel exclusively responsive to ATP [63], allowing Ca^2+^ and Na^+^ influx and K^+^ efflux. Each subunit has two membrane-spanning domains (TM1 and TM2) with an intracellular N-terminus and C-terminus, and most of the protein is extracellular with the ligand-binding region at the intersection of two subunits [63]. Regarding P2Y G-protein-coupled receptors, eight genes have been cloned in mammals (*P2RY1, P2RY2, P2RY4, P2RY6, P2RY11-14*). The preferred natural agonists are ATP (P2Y11), ADP (P2Y1, P2Y12, and P2Y13), UTP (P2Y2 and P2Y4), UDP (P2Y6), and UDP-sugars (P2Y14). P2Y1, 2, 4, and 6 act through Gq-phospholipase C β (PLCβ), producing inositol-triphosphate (IP3), which causes Ca^2+^ release from the endoplasmic reticulum, and diacylglycerol (DAG), which activates PKC. P2Y12-14 receptors act through Gi protein, causing adenylyl cyclase inhibition and a reduction in cyclic adenosine monophosphate (cAMP) levels; and P2Y11 receptor activates Gs protein, activating adenylyl cyclase and augmenting cAMP levels [64].

As molecular messengers, ATP and ADO can act as counterparts of each other, recognizing their specific receptors. For example, ATP is mostly a pro-inflammatory molecule, whereas ADO plays an important anti-inflammatory role. The fine-tuned regulation of these antagonist actions indeed depends on the set of ATP and ADO receptors expressed by the cellular system; they are also contingent on the activity of a set of extracellular hydrolytic enzymes that turn ATP into ADP, AMP, and ADO. For example, ATP can be converted into ADO in a two-step enzymatic process involving the CD39 (with apyrase-like action) and CD73 [65].

For a long time, it has been reported that purinergic signaling plays important roles in many physiological and pathological events. Haulica et al. reported in 1973 that, in addition to neurotransmission and blood coagulation, ADO exerts a hypnogenic action that was later confirmed [66]. Both ATP and ADO can act as coronary vasodilator agents [67]. Additionally, in the context of the orchestrated immune response, the alternative actions of ATP and ADO have been described [65]. Purinergic system malfunction contributes to the mechanisms of various illnesses, including cancer, diabetes, gout, osteoporosis, and cardiovascular, neurological, and psychiatric diseases [62, 68, 69].

## Purinergic signaling in cancer

Recent reviews highlight the diversity and plasticity of purinergic signaling in the context of cancer [70–73]. The purinergic system in the TME has a particular configuration that gives it a prominent role in cancer progression, with some notable characteristics: (1) cancer cells have a high capacity to produce ATP as a result of metabolic adaptations such as the Warburg effect, making cancerous cells energetically sustainable [74]; (2) ATP efflux to the interstitium is increased due to the boost in ATP synthesis, resulting in a high concentration of nucleotides (hundreds of µM) in the TME, which is enough to activate any purinergic receptor subtypes [39]; (3) purinergic receptors are widely expressed in tumor cells [75], and some subtypes, such as P2X7 receptor, are overexpressed in specific cancers [76–79]; and (4) the expression of ecto-nucleotidases (mainly CD73) contributes to regulating purinergic ligand concentrations in the TME [80]. CD39 and CD73 promote an immunosuppressive environment in the TME [81]. CD73, which is rate-limiting in the degradation of AMP into ADO, regulates tumor proliferation and progression and has therefore been defined as a prognostic marker for tumor survival [82].

The aforementioned characteristics suggest that the purinergic system is a fundamental element of the TME with a dual role. At the cellular social level, it mainly induces immunosuppression by mediating interactions with host immune cells. It also exerts autocrine-paracrine actions by directly regulating processes such as metabolism, cell proliferation, and cell migration and inducing epithelial-to-mesenchymal transition (EMT).

### The purinergic system at the cellular social level: from “find me” signaling to evasion of antitumor immune response

ATP has been categorized as a “find me” signal that triggers the immune response. In the cancer context, ADO—a direct product of ATP hydrolysis catalyzed by the sequential actions of CD39 and CD73 ecto-nucleotidases—elicits an immunosuppressive response by modulating the phenotype of tumor-infiltrated immune cells [83, 84]. Thus, the identity and proportion of purinergic ligands directly contribute to the balance of antitumor immune attack.

The *find me* role of ATP has been described in the context of tissue damage generated by conditions such as hypoxia, inflammation or necrosis. Under these conditions, ATP acts as a damage-associated molecular pattern (DAMP) [85], attracting dendritic cells, macrophages, and neutrophils to prompt damage resolution [86, 87]. In cancer conditions, especially when anti-cancer therapies induce cell death, extracellular ATP increments notably in the TME, and nucleotides can activate receptors in resident non-cancerous cells. For instance, in dendritic cells, ATP activates P2X7 receptors, inducing NLRP3 inflammasome assembly and the release of IL-1β, a proinflammatory cytokine with the capacity to induce an immunogenic response through the regulation of CD8^+^ T cells [88, 89]. On the other hand, actions through P2X and P2Y receptors can determine macrophage subpopulations that promote tumor development (tumor-associated macrophages), thereby establishing a protective TME for cancer cells and inducing CSC development and dissemination [90, 91].

Moreover, a consequence of the increase in extracellular ATP concentration is ADO accumulation in the TME as a result of ecto-nucleotidase activity [92]. ADO, acting through ADO receptor-dependent mechanisms, inhibits the antitumor immune response. Thus, after CL8-1 tumor melanoma cell xenotransplantation in mice, pharmacological inhibition or genetic deletion of the A2A receptor enhanced the inhibition of tumor growth, vascularization, and the destruction of metastases by incoming antitumor T lymphocytes [92], demonstrating that the ADO/A2A receptor pathway is essential for the modulation of immune host-tumor interactions. Furthermore, phenotypic modulation to inhibit the antitumor immune response via the ADO/A2A receptor pathway was documented for Tregs [93–95], T effector cells [95–97], natural killer cells [98, 99], and myeloid cells [100–102]. On the other hand, in medulloblastoma cells, CD79 overexpression reduced tumor proliferation, possibly by inducing differentiation and apoptosis through A1 receptor activation [82].

### Autocrine-paracrine actions of purinergic signaling in TME

As mentioned above, purinergic receptors are widely expressed in cancerous tissues [75], and ATP is available in the TME [39]; consequently, purinergic-mediated autocrine-paracrine communication regulates diverse physiological aspects in cancer cells, such as cell proliferation and cell migration.

P2X7 and P2Y2 receptors are two of the most studied purinergic receptors. Although P2X7 receptor was originally described as a cytotoxic receptor promoting cell death, it has been demonstrated to activate proliferative pathways in the TME, such as Ca^2+^-dependent and independent ERK phosphorylation [103–105], mTOR-HIF1-α-VEGF [106], and PI3K-AKT [107, 108], as well as tumor metastasis [109]. P2X7 can induce cell death and promote tumor proliferation and survival. These divergent actions have been related to two splice variants of the *P2RX7* receptor gene: the P2X7A and B isoforms. While the P2X7A variant comprises the full-length receptor, capable of pore formation with cytotoxic activity, the truncated P2X7B variant lacks the C-terminal tail and thus cannot form pores. However, ion channel opening and consequent downstream cellular signaling functions in this receptor contribute to the TME and promote tumor progression, metastasis, and chemotherapy resistance (reviewed by [110]). Tumor-promoting actions for P2X7 receptor have been described for mesothelioma [78], pancreatic carcinoma [105, 111], ovarian carcinoma [79], osteosarcoma cells [106], and neuroblastoma cells [109].

On the other hand, P2Y2 receptor is coupled to Gq heterotrimeric G-proteins, and its activation induces Ca^2+^ release from intracellular storage by the PLC/IP3 pathway [64]. P2Y2 activity has also been related to the activation of PI3K/Akt [112, 113], mitogen-activated protein kinases (MAPK) ERK [114–116] and JNK [116, 117], and mTOR kinase [118]. P2Y2 receptor-induced proliferation has been described for cancers in a broad group of tissues, such as the lung [119], breast [115, 120], ovary [121], cervix [122], liver [123], and stomach [124]. P2X7 receptor has been associated with the induction of cell migration and/or the triggering of EMT in the lung [125], breast [126, 127], prostate [128], and colorectal cancer cells [108]. P2Y2 receptor modulates these processes in the breast [115, 120, 129], prostate [128, 130], ovary [131], liver [123], stomach [132], and pancreas [133]. Thus, purinergic signaling constitutes a set of autocrine-paracrine signals that contribute to the regulation of multiple processes to support cancer progression.

## Purinergic signaling in CSCs

The implication of ATP and the purinergic system in nSCs is gradually becoming clearer. And while only a few studies have explored the role of the purinergic system exclusively in CSCs, extensive research has assessed the importance of purinergic receptors in stem-like traits that confer a malignant phenotype. Within tumor heterogeneity, CSCs are central to cancer phenotype regulation given their chemoresistance [134], enhanced DNA repair mechanisms [135], higher reactive oxygen species scavenging capacity [136], and metastatic competence [137]. In the next sections, we present some of the most recent literature concerning purinergic system components and their role in stem-like traits (summarized in Table 1).

**Table 1:** Purinergic elements important for a stem-like phenotype

Purinergic element	Effect	Model	Reference
A1, A2A, A2B, and A3	Augmented expression at mRNA and protein levels in CSCs (spheroids) derived from monolayers. Pharmacological antagonism downregulates CD133 and abolishes neurosphere formation. Pharmacological antagonism favors temozolomide-induced cell death	U87MG and U343MG glioblastoma multiforme cell lines	[138]
A2A	Agonist induces activation of PI3K-Akt-mTOR pathway. Induction of Nanog, OCT-4, SOX-2 and CD44. Activation favors radioresistance	MKN-45 gastric cancer cell line	[139]
A2B and A3	Antagonism decreases MRP3 (multidrug resistance protein) in hypoxia. Antagonism and knockdown (using siRNA) increases sensitivity to chemotherapy (teniposide) in hypoxia	U87MG glioblastoma multiforme cell line	[140]
A2B	Spheroids present higher transcript levels. Agonist activation promotes spheroid formation derived from monolayer cultures. Pharmacological activation in CSCs induces apoptosis	MCF7 and MDA-MB-231 breast cancer cell lines	[141]
A2B	Overexpression in isolated CSCs. Knockdown impedes spheroid formation. Exogenous adenosine augments proliferation in CSCs and tumor formation (in mice)	U87 and GL1 glioblastoma multiforme cell lines	[142]
A2B	In hypoxic conditions, antagonism reduces EMT markers (*SNAIL*, *TWIST*, *vimentin* and *CDH-2*)	U87MG, GB,38, and GBM27 glioblastoma multiforme cell lines	[143]
A2B	Overexpression in mammospheres. Agonist (adenosine) increases ALDH activity and antagonism (caffeine) does the opposite	MCF7 and SUM149 breast cancer cell lines	[144]
A2B	Overexpression in chemotherapy-resistant cell lines. Up-regulation in mammospheres from the monolayer. Knockdown reduces spheroid formation and ALDH activity and downregulates transcript levels of *NANOG*, *SOX2*, and *KLF4*	MDA-MB-231, SUM149, and SUM169 breast cancer cell lines	[144]
CD73	Overexpression in spheroids. Higher production of adenosine, compared to the monolayer. Adenosine promotes TGF $\beta$ expression which enhances CD73 expression	CaSki cervical cancer cell line	[145, 146]
CD73	Overexpression in spheroids. Higher production of adenosine, compared to the monolayer	H2228, H3122, and A549 NSCLC cell lines	[147]
CD73	Overexpression in spheroids. Knockdown decreases spheroid formation	NOZ gall bladder cancer cell line	[148]
CD73	Knockdown using nanoparticles carrying siRNAs favor chemotherapy	4T1 breast cancer cell line	[149]
CD73	Knockdown using nanoparticles carrying siRNAs favors sensitivity to temozolomide	C6 and U138MG glioblastoma multiforme cell line	[150]
CD73	High protein levels correlated to stem-like markers (SOX2, CD44, ALDH) and EMT markers (*N-Cadherine*, *vimentin*, *Notch*)	Patient-derived high-risk stage 4 neuroblastoma cell lines (CHLA-20 and CHLA-90)	[151]
P2X7	Agonist activation upregulates EMT markers in mRNA (*CDH2*, *SNAIL*, *Zeb1*) and protein (N-cadherin, ZEB1, vimentin, TWIST). SMAD2 phosphorylation. Effects counteracted by P2X7 antagonist (A438079)	Patient-derived glioblastoma stem cells	[152]
P2X7	Overexpressed in leukemia-initiating cells and leukemia granulocyte-monocyte progenitors. Knock-out decreased homing and invasiveness	Murine-induced AML model	[153]
P2X7	Activation increases EMT markers (vimentin, N-cadherin). Agonist administration promotes tumorigenesis and metastasis	HOS/MNNG osteosarcoma cell line. Xenograft tumor in mice	[106]
P2X7	Synthetic antagonists inhibit spheroid formation	TS15-88 glioblastoma cell line	[154]
P2X7	Activation augments EMT markers (vimentin, snail, and fibronectin)	SW-620 and HCT-116 colorectal cancer cell lines. Xenograft tumor in mice	[108]
P2Y2	High protein levels correlate with Notch-4	Patient-derived breast cancer cells	[155]
P2Y4	Activation induces neurite differentiation. Transient overexpression favors differentiation to neurites	SH-SY5Y neuroblastoma cell line	[156]
P2Rs	Caspase-mediated apoptosis induced by ATP. Increased sensitivity to chemotherapy (Ara-C) by ATP	CD34 + CD38- from AML patient-derived cells	[157]
P2Rs	Decreases viability by exogenous ATP, counteracted by P2 antagonists. Increased sensitivity to temozolomide using exogenous ATP	Patient-derived glioblastoma stem cells	[158]

### ATP

One of the best-characterized systems in stem cell biology is the hematopoietic system, as well as the role of purinergic system in stem cell differentiation and pool regulation in hematopoiesis. As Paredes-Gamero and coworkers have revised [159], P2 receptors are differentially expressed in the different stages of hematopoiesis, and ATP is released by different cells in the hematopoietic niche (endothelial cells and osteoblasts). ATP may act as a regulator for hematopoietic stem cells (HSCs). One study reported that ATP increased a CD34^+^ cell population from adult healthy donors [160], while another study showed how ATP reduced the percentage of HSCs and myeloid progenitors [161]. Although the effect of ATP on stem cell physiology might seem contradictory, its influence on HSCs is evident.

As shown previously with nSCs, ATP might regulate CSC populations. In a primordial study published in 2012, the addition of exogenous ATP to glioblastoma cell lines (U87, C343, and C6) reduced spheroid size and numbers in the spheroid-formation assay and lessened the expression of the stem cell markers CD133 and OCT-4 [162]. In a similar way, ATP decreased the viability of patient-derived CSCs and increased their sensitivity to chemotherapy in glioblastoma [158] and acute myeloid lymphoma [157], thus implying that ATP can impact CSC survival. On the other hand, purinergic signaling could promote chemoresistance as ATP hydrolysis by CD73 enhanced temozolomide cytotoxicity in the glioblastoma cell lines M059J and U251, thereby supporting cell cycle arrest and cell death [163].

### CD73

**Fig. 2:**
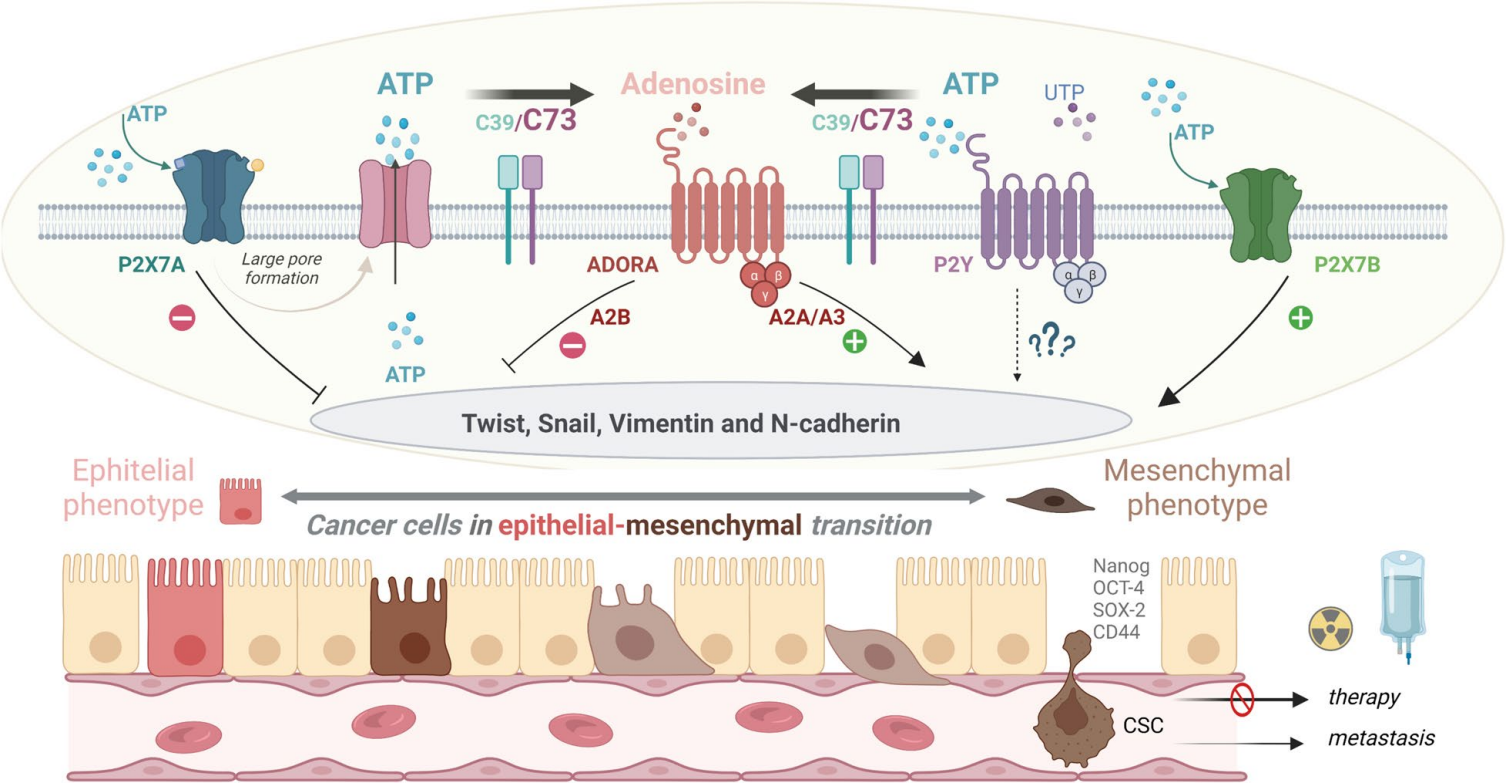
Purinergic signaling in the process of the epithelial-mesenchymal transition in cancer cells. Epithelial-mesenchymal transition (EMT) involves the transformation of epithelial cells into mesenchymal-like cells, characterized by the loss of epithelial characteristics and the acquisition of mesenchymal traits. This transition is associated with increased migratory capacity, invasiveness, and the development of stem-like properties, leading to the formation of cancer stem cells (CSCs). CSCs are resistant to standard cancer treatments and contribute to tumor recurrence and metastasis. Purinergic signaling encompasses signaling pathways mediated by purine nucleotides, such as adenosine triphosphate (ATP), as well as its breakdown products, such as adenosine, facilitated by the ectoenzyme CD73. These signaling molecules interact with specific purinergic receptors expressed on the cell surface, including ADORA, P2X, and P2Y receptors. Extensive research has implicated purinergic signaling in the regulation of EMT and the maintenance of CSCs. Notably, the P2X7 receptor has emerged as a key player in this process. Activation of P2X7 isoform B has been associated with the induction of EMT and the expression of EMT-related transcription factors, such as TWIST and SNAIL, as well as mesenchymal markers like vimentin and N-cadherin. Additionally, P2X7 receptor activation has been linked to the acquisition of stem-like properties by cancer cells. Another important component of the purinergic signaling pathway involved in EMT and presence of stemness markers (SOX2, CD44 and ALDH) is CD73, an ectoenzyme responsible for generating extracellular adenosine. Adenosine can activate specific adenosine receptors, including ADORA A2A and A3 receptors, which have been shown to promote EMT and the expression of EMT markers. While the role of P2Y receptors in EMT is not well-studied, the activation of P2X7 isoform A and ADORA A2B receptors appears to counterbalance the EMT process. Overall, modulating purinergic signaling pathways, particularly by targeting P2X7 isoform B, CD73, and ADORA A2A and A3 receptors, shows potential as a therapeutic strategy to inhibit EMT, disrupt the stem-like properties of cancer cells, and enhance the effectiveness of cancer treatments. Created with BioRender.com

Ecto-5′-nucleotidase (NT5E/CD73) is one of the most extensively studied components of the purinergic system and a highly important regulator of extracellular ATP concentration. Thus, it is not surprising that there is information about its role in CSCs. High expression of CD73 has been correlated with stemness markers in different models. For example, in cell lines derived from patients with stage 4 neuroblastoma, CD73 protein levels were correlated with the presence of stemness markers (SOX2, CD44, and ALDH) and transcripts of EMT markers (N-cadherin, vimentin, Notch) [151] (Fig. 2). In another study, hepatocellular carcinoma cell lines were sorted based on CD73 expression levels. According to its findings, CD73^High^ had a high expression of the stemness marker SOX9 and a high capacity for spheroid formation. However, when CD73 was knocked down, SOX9 was downregulated, and spheroid formation was abolished [164]. In addition, a renal carcinoma cell line (786-O) was sorted in a similar way, and the CD73^High^ population upregulated *Oct-3/4* expression and was enriched in mytomycin C-resistant cells [165], which was not observed in the CD73^low^ population. In this same study, CD73 was evaluated in spheroids derived from 786-O cell lines, and higher levels of the ecto-nucleotidase were found in contrast with the monolayer culture.

Other studies have shown that spheroids derived from cancer cell lines have a higher CD73 expression than their parental cell line. In a cervical cancer cell line (CaSki), CD73 was increased in spheroids in contrast to their monolayer cultures, thus promoting a higher extracellular concentration of ADO. The authors of the study mention the plausible positive feedback loop between ADO and CD73, given that ADO promotes TGF-$\beta$ expression, which further enhances CD73 expression [145, 146]. To strengthen these findings, Bertolini and coworkers identified CD73 overexpression in spheroids from NSCLC cell lines and a higher production of ADO [147].

CD73 knockdown has been evaluated as well, and similar results have been found in different models. In a gall bladder cancer cell line NOZ, spheroids presented a high expression of CD73 and knockdown using siRNA impeded spheroid formation [148]. Furthermore, in ovarian cancer cell lines and fresh tumor tissue, CD73 silencing using siRNA downregulated EMT markers and spheroid formation capacity [166]. Other studies have evaluated chemotherapy resistance, as it is a well-established stem-like trait. Chemotherapy resistance was shown to be impeded after CD73 knockdown using siRNAs in both glioblastoma and breast cancer cell lines [149, 150]. Moreover, CD73 silencing using siRNA-containing nanoparticles in murine cancer cell lines from colon, breast, and melanoma diminished cell migration, proliferation, and resistance to doxorubicin [167].

### Adenosine receptors

ADO is present in high concentrations in the TME, and its relevance as an immune modulator can be reviewed elsewhere. Here, we will focus on the role of adenosine receptors (*ADORA*) in stem-like properties. In glioblastoma cell lines, there is homogenous information showing that adenosine receptors A1, A2A, A2B, and A3 are overexpressed in spheroids and are correlated with stemness markers (CD133), EMT markers (*vimentin*, *SNAIL*, *TWIST,* and *CDH1*), and pharmacological antagonism. Knockdown of this receptors using siRNAs abolishes spheroid formation capacity, increases the sensitivity to chemotherapy, and impedes tumorigenesis [138, 140, 142, 168, 169]. In breast cancer, there is clear evidence that A2B receptor is correlated with a stem-like phenotype, since A2B is overexpressed in spheroids from different cell lines (MCF7, MDA-MB-231, SUM149, and SUM169) and its pharmacological activation in such CSCs enhances ALDH activity. On the other hand, A2B knockdown diminishes spheroid formation capacity and stemness markers (*NANOG*, *SOX2*, *KLF4,* and ALDH activity) [141, 143, 144]. To reinforce the importance of ADO receptors in CSCs, one study evaluated A2A in a gastric cancer model (MKN-45 cell line) and showed that receptor activation induced transcription factors that are essential for stemness (Nanog, OCT-4, SOX2, and CD44) and favored radioresistance [139] (Fig. 2).

### P2Y receptors

In comparison to the other elements of the purinergic system, P2Y receptors have been poorly assessed in CSCs. In a neuroblastoma model (SH-SY5Y cell line), P2Y4 receptor pharmacological activation induced differentiation to neurites, as did transient overexpression [156]. Although P2Y2 receptor is one of the most interesting targets for cancer research, there is only one paper evaluating, albeit tangentially, its role in stem-like properties. In patient-derived breast cancer cells, P2Y2 receptor levels were correlated with the stemness marker Notch-4 [155]. The lack of information on the topic of P2Y receptors, CSCs, and stem-like traits in cancer is evident.

### P2X7 receptor

P2X7 receptor, which induces tumor proliferation, contributes to the maintenance of embryonic stem cell pluripotency [170, 171]. And similar functions are expected in maintaining a pool of CSCs capable of causing tumor relapse and chemoresistance [172]. A piece of evidence points to P2X7 receptor as a key player in metabostemness, the metabolic reprogramming of cancer cells toward an undifferentiated phenotype (i.e., CSCs). P2X7 receptor modulation is associated with metabolic targets closely related to cellular events for stemness phenotype acquisition [173]. Importantly, Lameu and coworkers have focused on the functions of P2X7 receptor and its isoforms in promoting the phenotypic transition of the tumor into a stemness stage and EMT. They have observed that P2X7A is important for triggering CSC differentiation, since knockdown cells for this isoform remain in an undifferentiated state. In contrast, P2X7B activation is implicated in EMT [110, 172], promoting treatment resistance and metastatic formation.

Similar to P2Y receptors, P2X7 receptor involvement in CSCs has been studied solely in a tangential way. In this context, using glioblastoma models, P2X7 activation has been related to the acquisition of EMT markers in mRNA (*CDH2*, *SNAIL*, *Zeb1*) and proteins (N-cadherin, ZEB1, vimentin, and TWIST) [152], as well as the promotion of spheroid formation [154]. Other cancer models, such as osteosarcoma and colorectal cancer cell lines, have demonstrated similar effects, where P2X7 activation increased EMT markers both at mRNA and protein levels [106, 112]. Yet, there is a study evaluating the presence of P2X7 receptor in different leukemia-initiating cells. This study used an induced murine model of acute myeloid leukemia, and P2X7 receptor was detected at higher levels in leukemia-initiating cells and granulocyte-monocyte progenitors [153]. Although the latter findings are remarkable, to our knowledge, there are no other papers characterizing P2X7 or their function in CSCs.

### Purinergic system and EMT

Thus far, we have described several purinergic elements and their implication in CSC biology. To elaborate on the purinergic system’s involvement in the stem-like phenotype, we will discuss how purinergic signaling modulates one important transdifferentiation program that has been shown to be continuously active in stem cells: EMT.

EMT is a transdifferentiation program in which epithelial cells gain mesenchymal characteristics, and it is involved in embryonic development, tissue repair, and cancer cell migration. EMT has been related to stem-like properties. Mammary epithelial cells undergoing EMT exhibited an amplified stem-like phenotype in terms of spheroid formation, soft agar colonies, and tumorigenic properties [174].

Considering that EMT confers stem-like properties, we focused on papers concerning the importance of the purinergic system in EMT. First, a study revealed that, in glioblastoma cell lines, hypoxia favored CD73 and ADO A3 receptor expression, which is notable considering the hypoxic conditions within the tumor. The study also showed that A3 receptor pharmacological inhibition decreased EMT markers such as TWIST, SNAIL, vimentin, and N-cadherin in such cell lines [168] (Fig. 2). With respect to P2 receptors, in colorectal cancer cell lines, pharmacological activation of P2X7 receptor led to EMT activation, as demonstrated by augmented vimentin, Snail, and fibronectin expression and decreased E-cadherin expression [108]. In neuroblastoma, a counterbalance of P2X7 isoforms was observed to promote EMT, pointing to an epithelial-prone P2X7A-related effect and P2X7B as an EMT-favoring isoform [172] (Fig. 2).

CSCs are highly resistant to chemotherapy and radiotherapy. Therefore, the paper published by Nguyen and coworkers is quite fascinating. According to the authors, a radioresistant pancreatic cancer cell line favored a mesenchymal state, which showed downregulation in E-cadherin and upregulation in vimentin and, surprisingly, CD73. In this same study, CD73 expression interference with shRNA led to radiosensitivity and an epithelial phenotype, evidencing the importance of CD73 in acquiring a mesenchymal phenotype [175].

## Concluding remarks

ATP and ADO in the purinergic system are important for the regulation of tumor cell proliferation and malignant progression, as well as for modulating the immune response and TME biology. There is abundant information highlighting the importance of the purinergic system in nSCs but not in CSCs, even when it has been proven that the system occurs in healthy conditions and is relevant in cancer pathologies. ATP depletion by CD73 activity might be involved in stem-like properties, but it raises the question of whether ATP depletion or ADO generation creates such a phenotype. Furthermore, conclusions should be drawn with caution, given that the phenotype produced by purinergic receptor activity depends on the tissue in question, the receptor being studied, and the experimental conditions. Diverse elements of the purinergic system have been shown to play essential roles in maintaining stemness, especially CD73, A2B, and P2X7 receptor, yet there is an obvious lack of understanding about P2Y receptors. The purinergic system is undoubtedly a promising field of knowledge for comprehending CSC biology.

## Data Availability

Not applicable.
